# The Association between Breast Tissue Optical Content and Mammographic Density in Pre- and Post-Menopausal Women

**DOI:** 10.1371/journal.pone.0115851

**Published:** 2015-01-15

**Authors:** Kristina M. Blackmore, Julia A. Knight, Jane Walter, Lothar Lilge

**Affiliations:** 1 Lunenfeld Tanenbaum Research Institute, Mount Sinai Hospital, Toronto, Ontario, Canada; 2 Princess Margaret Cancer Centre and Department of Medical Biophysics, University of Toronto, Ontario, Canada; Taipei Medical University, TAIWAN

## Abstract

Mammographic density (MD), associated with higher water and lower fat content in the breast, is strongly related to breast cancer risk. Optical attenuation spectroscopy (OS) is a non-imaging method of evaluating breast tissue composition by red and near-infrared light transmitted through the breast that, unlike mammography, does not involve radiation. OS provides information on wavelength dependent light scattering of tissue and on absorption by water, lipid, oxy-, deoxy-hemoglobin. We propose that OS could be an alternative marker of breast cancer risk and that OS breast tissue measures will be associated with MD. In the present analysis, we developed an algorithm to estimate breast tissue composition and light scattering parameters using a spectrally constrained global fitting procedure employing a diffuse light transport model. OS measurements were obtained from 202 pre- and post-menopausal women with normal mammograms. Percent density (PD) and dense area (DA) were measured using Cumulus. The association between OS tissue composition and PD and DA was analyzed using linear regression adjusted for body mass index. Among pre-menopausal women, lipid content was significantly inversely associated with square root transformed PD (β = -0.05, p = 0.0002) and DA (β = -0.05, p = 0.019); water content was significantly positively associated with PD (β = 0.06, p = 0.008). Tissue oxygen saturation was marginally inversely associated with PD (β = -0.03, p = 0.057) but significantly inversely associated with DA (β = -0.10, p = 0.002). Among post-menopausal women lipid and water content were significantly associated (negatively and positively, respectively) with PD (β_lipid_ = -0.08, β_water_ = 0.14, both p<0.0001) and DA (β_lipid_ = -0.10, p<0.0001; β_water_ = 0.11, p = 0.001). The association between OS breast content and PD and DA is consistent with more proliferation in dense tissue of younger women, greater lipid content in low density tissue and higher water content in high density tissue. OS may be useful for assessing physiologic tissue differences related to breast cancer risk, particularly when mammography is not feasible or easily accessible.

## INTRODUCTION

Mammographic density has been strongly associated with breast cancer risk [1–13] and has also been related to other known risk factors for the disease [10]. Radiologically, fat has a lower x-ray attenuation coefficient than fibroglandular tissue and appears darker on a radiograph, whereas regions of brightness characterize stromal and epithelial tissue and are referred to as mammographic density [13]. Quantitative assessment of percent density (PD) has shown that women with dense tissue occupying ≥ 75% of the total breast area have a 4 to 6 fold higher risk of being diagnosed with breast cancer during the next decade than women in the lowest category (< 10%) [1–13]. In some studies, the dense area (DA, cm^-2^) has also been shown to be associated with breast cancer risk [14, 15].

Mammography is commonly used to assess breast tissue density; however, it may not be suitable for use in etiology studies involving young women or women at increased risk (e.g. mutation carriers) or in prevention studies requiring frequent monitoring because it involves exposure to ionizing radiation. A potential alternative is optical spectroscopy (OS), a non-imaging method of evaluating parts of the biochemical composition of breast tissue that involves no exposure to ionizing radiation and no breast compression [16–20]. Red and near-infrared (NIR) light in the spectral range of 650 to 1000 nm illuminates the breast at < 200 mWcm^-2^ power density. This range was selected because the resulting transmission spectra provide information on the wavelength dependent light scattering of the tissue and on the absorption by four dominant chromophores, namely water, lipid, oxy- and deoxy-hemoglobin, from the latter two which total hemoglobin content (THC) and oxygen saturation (S_t_O_2_) can be derived [21–25]. A limited number of optical studies with small sample sizes have shown increased water and total hemoglobin content, decreased lipid content and lower oxygen saturation with increasing density categories according to BIRADS classification [26–29]. One study showed a positive correlation between water and hemoglobin optical content and an inverse correlation between lipid optical content and MRI assessed breast density [29].

In a previous cross-sectional study of 225 pre- and post-menopausal women without radiologically suspicious lesions we showed that OS scores derived from principal component analysis (PCA) of the diffuse transmission spectra were associated with PD and DA [19]. However, analysis of breast tissue composition using OS scores has its limitations, as the concentrations of the dominant chromophores cannot be quantified but only inferred from their contribution to the overall spectral shape of the principal component spectra, which generally contain contributions from all chormophores. As a result, we developed an algorithm to quantify the optical content of breast tissue using a diffuse light transport model. Here we report on the relationship between OS derived concentrations of water and lipid, as well as THC and S_t_O_2_, and light scattering parameters (power and amplitude) with PD and DA in the same sample of women. Compared to previous studies relating optically derived tissue composition measures to mammographic density [26–29], our study includes a larger sample of both pre- and post-menopausal women (n = 95 and 107, respectively) with normal mammograms, and examines results for both PD and DA measured quantitatively.

## MATERIALS AND METHODS

### Study Population

As previously described [16–20], study participants were recruited between March 1, 2000 and September 30, 2004 from the Marvelle Koffler Breast Imaging Centre at Mount Sinai Hospital in Toronto, Canada. Written informed consent was obtained from all women who participated. All procedures used in this study, including the consent process, were approved by the Research Ethics Boards of the University of Toronto, Mount Sinai Hospital and the University Health Network. Inclusion criteria required an available analogue (film) standard screening mammogram within thirteen months prior to recruitment revealing no radiological suspicious lesions (N = 232), based on assessment by 2 independent radiologists. Exclusion criteria were prior fine needle aspiration, core biopsies or other type of breast surgery including breast reduction or augmentation and breast tattoos.

Participants’ age, menopausal status, height and weight were collected by a self-administered questionnaire. Post-menopausal status was defined as having had no menstrual period for at least 12 months prior to mammography. One woman who classified herself as pre-menopausal was excluded given her age was 59 years. An additional 14 pre-menopausal and 15 post-menopausal women were also excluded due to either poor spectral fitting (fit error > 27, see below) and/or because the total optical content (%) accounted for by the major tissue components (lipid, water and THC) was ≤ 57%. Hence the data presented here are from 202 women (95 pre-menopausal and 107 post-menopausal) with complete spectral and demographic information.

### Optical Set-Up and Procedure

The instrumentation used to gather attenuation spectra was described previously in detail [16–20]. A 50W halogen lamp served as broadband light source. The radiance emitted by a 5 mm diameter liquid light guide (Fibre Guide, Bridgeport, CT) placed in contact with the skin on top of the breast was below 200 mW and covered the spectral range of 550 nm to < 1300 nm. Contact with the breast was made with minimal compression as not to displace blood and other liquids beneath the source. Transmitted light was collected via a ~ 3 mm diameter optical fibre bundle (comprising 140 Si/Si fibres, 200 µm core diameter fibers, with a numerical aperture of 0.36, P & P Optica, Kitchener, Canada) held coaxially and pointing towards the light source. A calliper provided the physical inter-optode distance in centimeters. A spectro-photometer (Kaiser, California, USA) with holographic transillumination grating (15.7rules/mm blazed at 850nm) and a 2D cryogenically cooled silicon CCD (Photometrics, New Jersey, USA) provided wavelength dependent detection between 625 nm and 1060 nm (n = 436 wavelengths, resolution 3 nm). Hospital Grade Canada Standards Association certification and Health Canada Investigational New Device Class II approval were obtained for use of the instrument on volunteers. The irradiance (Wcm^-2^) used was well below the 2001 IEC 60825–1 guidelines for exposure of skin to light [30, 31].

All OS measurements were collected prior to quantification of mammographic features (PD, DA). Previous analyses showed no evidence of an effect of the length of time between mammography and OS measures on their association [19]. Also, for pre-menopausal women all measurements were taken roughly during the first week of the luteal phase of the menstrual cycle based on the woman’s self-reported last menstrual bleed and usual cycle length over 2 or more consecutive cycles. A previous repeated measures analysis in a subset of pre-menopausal women over a 4-week period showed high reproducibility between optical spectra taken weekly and no significant difference in the optical spectra over menstrual cycle phase (follicular versus luteal) [19, 20].

Transmission measurements were taken in the dark, with the participant seated upright and the breast resting on the support platform. Optical light attenuation measurements were executed in four standardized positions (center, medial, distal, lateral) on each breast (left and right) in cranial caudal view (n = 8 transmission spectra), providing optical interrogation of different anatomical regions [16–19]; however, in the present analysis only OS measurements at the distal position on each breast, located 2cm posterior to the nipple, were used. Measurements at this location allow interrogation of the ductal and lobular complex (i.e. fibroglandular tissue). This location also provides the best true absorption spectra as the medial and lateral positions are more prone than the distal position to light losses at the breast boundary adding complexity to interpretation of the measured attenuation spectra in terms of absorption. Equally the center position, especially in smaller breasts, also includes optical interrogation of the pectoral muscle and chest wall, resulting in incorrect spectral fitting due to absorption by myoglobin.

### Spectral Estimation of Tissue Optical Properties

**Figure 1:**
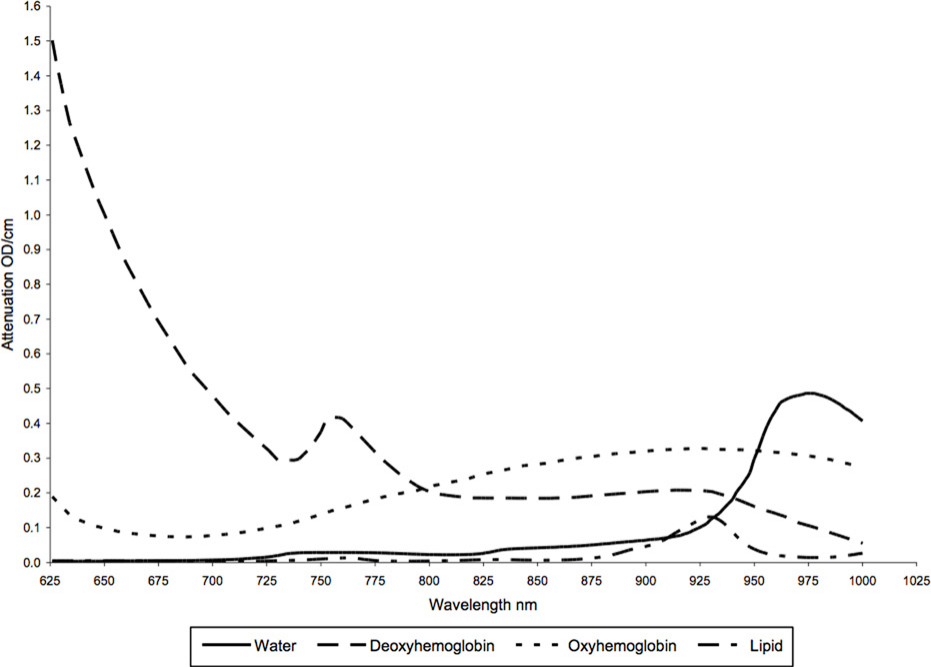
The attenuation spectra (optical density (OD) per cm over all wavelengths) of four main breast tissue components—water, lipid, oxyhemoglobin, and deoxyhemoglobin.

For each woman, transmission spectra were first converted into attenuation spectra (OD cm^-1^) using an optical system throughput calibration standard and corrected for dark noise [16–19]. A spectrally constrained global fitting procedure based on the previously published diffuse approximation of light transport in tissue [32], was used to estimate the concentrations of deoxy-hemoglobin (*µ*M), oxy-hemoglobin (*µ*M), lipid (%), water (%), as well as two light scattering parameters, scattering amplitude (µ_a_, cm^-1^) and wavelength-dependent scattering power (b), for all measured positions. Standard absorption spectra for the four dominant chromophores of interest were obtained from the literature [33–35] (Fig. 1). The reduced light scattering coefficient (µ_s_’cm^-1^) was modeled using an exponential function of wavelength (λ) given by µ_s_’(λ) = *a*(λ)^-*b*^, where *a* and *b* are free fitting parameters, as defined above [36]. Hence, in total there were six free parameters, four chromophore concentrations and two scattering parameters, used in the fitting algorithm.

Because of the use of the diffusion model for infinite slab geometry, constraints were placed on the solution space to eliminate physically and biologically unrealistic results, due to compensation for light losses at the breast boundary through excessive absorption, and a penalty was added to the fit if the total was in excess of 100% (Table 1). Four starting conditions were selected for each parameter based on results obtained from the literature [21–25] to avoid under-sampling the full parameter space with the fitting algorithm and converging to local minima. The absorption of each chromophore and reduced light scattering coefficients (a, b) at each position were obtained by chi-square (*X^2^*) minimization of the difference between the measured and calculated spectrum (i.e. fit error) according to eq. 1: *X^2^ = Σ_λ_√(y_t_(λ)-y_fit_(λ))^2^/*y_t_(λ)^2^, where λ is the wavelength (from 625 to 1060nm), y_t_(λ) is the measured spectrum and y_fit_(λ) is the spectrum calculated using the diffusion theory approximation [32]. The fit with the lowest *X^2^* value was used for the final chromophore and light scattering parameter results. Composite indices of tissue metabolism and vascularization were also calculated; total hemoglobin concentration (THC, *µ*M) was calculated as the sum of deoxy- and oxy hemoglobin and tissue hemoglobin oxygen saturation (S_t_O_2_, %) was calculated as the ratio of oxy-hemoglobin to total hemoglobin multiplied by 100%.

**Table 1:** Constraints and penalties employed in fitting algorithm used to obtain chromophore concentrations and scattering parameters.

**Optical Parameter**	**Range of Values**	**Type of Constraint**
Total Hemoglobin Concentration (*µ*M)	≥ 0 *µ*M, ≤ 56 *µ*M	Rigid Constraint^1^
Hemoglobin Oxygen Saturation (S_t_O_2_, %)	≥ 0%, ≤ 100%	Rigid Constraint
Lipid Concentration (%)	≥ 0%, ≤ 100%	Rigid Constraint
Water Concentration (%)	≥ 0%, ≤ 100%	Rigid Constraint
Scatter Amplitude (cm^-1^)	≥ 1cm^-1^, ≤ 40cm^-1^	Rigid Constraint
Scatter Power	≥ 0.05, ≤ 5.0	Rigid Constraint
Sum of Chromophore Concentrations (%)	≥ 0%, ≤ 100%	Penalty^2^
Amplitude of Best-Fit Spectrum	> Amplitude of Participant Spectrum	Penalty

^1^Rigid constraints are restrictions on the fit such that if the value being constrained is out of range, those fit parameters are rejected by the algorithm. The fit algorithm will go back to the previous set of fit parameters and will continue the search for a minimum in another direction

^2^Penalties are constraints where if the value being constrained is out of range, a fixed penalty value is added to the *X* ^2^ value for each instance where the fit value is out of range to artificially indicate that the fit is not good

### Quantification of Percent Density and Dense Area from Mammograms

All film mammograms in cranial-caudal view [n = 2 films (left and right) x 202 eligible women] were digitized using a Lumisys Digital Scanner (Kodak, New York, USA) with 12-bit gray scale resolution and a pixel spacing of 260 μm/pixel. PD [(dense area/total area) x 100] and DA were estimated from the digitized mammogram using the computer assisted thresholding program Cumulus [6, 37]. Dense area was converted into centimeters squared (cm^2^) using an area of 0.000676 cm^2^ for each pixel. All images were read by one expert and two trained individuals (KMB and LL), with each mammogram repeated twice by each reader with a period of at least one month separating each read. The reproducibility of mammographic measurements on duplicate readings (15% random repeat set) was high; the resulting intra-class correlation coefficients for the two trained readers for the final read were 0.96 and 0.93, respectively. Agreement between each trained reader and an expert for the final read was also high with interclass correlation coefficients of 0.92 and 0.93, respectively.

### Data Analysis

Each OS breast tissue optical parameter at the distal position (THC, S_t_O_2_, lipid, water, scattering amplitude and power) was averaged over both breasts. Bilateral symmetry in the optical spectra at the distal position was previously demonstrated [16]. Given the high correlation between readers and between readings from the right and left breasts (r > 0.90), which is expected in normal symmetric development of the bilateral organ, the final Cumulus results were averaged for each woman over the three readers (2 trained and 1 expert) over both breasts. The correlation between left and right PD and DA in the population under study was also high (r_PD_ = 0.97 and r_DA_ = 0.90).

We first assessed the Pearson correlations between each OS breast tissue parameter and age and BMI and with each other, separately among pre- and post-menopausal women. To measure the association between each OS breast tissue parameter (THC, S_t_O_2_, lipid, water, scattering amplitude and power) and each mammographic density outcome (PD and DA) we employed univariate and multivariate linear regression analysis using backwards stepwise elimination. As PD and DA were not normally distributed, we used a square root transformation, which resulted in more normal distributions. The distributions among the majority of independent variables (age, BMI and the OS breast components with the exception of scattering power which was slightly skewed) were reasonably normally distributed for both pre- and post-menopausal women and hence no transformations were performed. As BMI and age were considered potential confounders, we looked at each OS breast parameter both unadjusted and adjusted for continuous age and BMI (kgm^-2^). Analyses were performed separately for pre- and post-menopausal women as we found significant interactions between menopausal status and S_t_O_2_ (p = 0.006), lipid content (p = 0.046) and water content (p = 0.021) for PD and between menopausal status and THC (p = 0.022), lipid content (p = 0.008) and water content (p = 0.01) for DA. Statistical analyses were carried out using SAS (Statistical Analysis Systems; SAS Institute Inc., USA) version 9.1 and p values < 0.05 were considered significant.

## RESULTS

As expected, pre-menopausal women had significantly higher PD and larger DA (both at p < 0.0001) compared to post-menopausal women (Table 2). As also shown in this table, THC, water, scattering amplitude, and, marginally S_t_O_2_, were also higher in pre-menopausal women while lipid content was lower.

**Table 2:** Means and standard deviations (SDs) of percent mammographic density, dense area, age, BMI and OS tissue optical properties by menopausal status.

	**Pre-menopausal (*N* = 95)**	**Post-menopausal (*N* = 107)**
Age (years) mean ± SD (Range)	45.7 ± 3.8 (37.0–54.0)	55.1 ± 6.2 (42.0–74.0)
		
BMI (kgm^-2^) mean ± SD (Range)	26.3 ± 6.5 (18.0–54.9)	26.8 ± 5.1 (18.6–43.9)
		
Percent Density (%) mean ± SD (Range)	34.7 ± 19.2 (1.3–79.5)	22.0 ± 17.3 (0.2–61.2)
		
Dense Area (cm^2^) mean ± SD (Range)	43.0 ± 26.3 (0.0–113.3)	28.0 ± 23.0 (0.1–101.3)
		
THC (*µ*M) mean ± SD (Range)	15.7 ± 4.8 (4.9–30.9)	12.4 ± 4.7 (4.0–27.1)
		
S_t_O_2_ (%) mean ± SD (Range)	70.5 ± 7.6 (49.0–93.7)	68.5 ± 8.0 (36.0–84.7)
		
Lipids (%) mean ± SD (Range)	61.5 ± 10.2 (36.0–80.6)	68.3 ± 9.0 (40.2–87.5)
		
Water (%) mean ± SD (Range)	21.3 ± 7.2 (10.1–44.5)	18.2 ± 5.9 (5.6–41.7)
		
Scattering Amplitude (cm^-1^) mean ± SD (Range)	14.6 ± 3.4 (6.9–24.2)	12.9 ± 2.9 (7.3–21.6)
		
Scattering Power mean ± SD (Range)	0.35 ± 0.31 (0.06–1.64)	0.38 ± 0.26 (0.06–1.25)

BMI; Body mass index

THC; Total hemoglobin content

S_t_O_2;_ oxygen tissue saturation

Among all women, BMI correlated positively with lipid content and negatively with water content, while age was significantly correlated to THC (negative) and lipids (positive) among post-menopausal women only (Table 3). Among the OS parameters, water and lipid content were negatively correlated with each other in both groups. S_t_O_2_ was also inversely correlated to water, although the correlation was stronger in pre-menopausal women, while THC was inversely correlated to lipids, but the correlation was stronger in post- menopausal women.

**Table 3:** Results of Pearson correlation analysis of OS parameters with age, BMI and each other among pre- and post-menopausal women.

	**Pre-Menopausal (*N* = 95)**				**Post-Menopausal *(N* = 107)**			
	**THC (µM)**	**S_t_O^2^ (%)**	**Lipids (%)**	**Water (%)**	**THC (µM)**	**S_t_O^2^ (%)**	**Lipids (%)**	**Water (%)**
Age (years)	-0.061 (0.558)	-0.007 (0.944)	0.118 (0.256)	-0.034 (0.743)	-0.353 (0.0002)	-0.130 (0.181)	0.324 (0.0007)	-0.028 (0.772)
BMI (kgm^-2^)	-0.094 (0.368)	0.424 (<0.0001)	0.266 (0.01)	-0.474 (<0.0001)	-0.188 (0.055)	0.052 (0.598)	0.275 (0.005)	-0.401 (<0.0001)
THC (µM)	-	0.287 (0.005)	-0.375 (0.0002)	0.103 (0.318)	-	0.239 (0.013)	-0.517 (<0.0001)	0.258 (0.007)
S_t_O^2^(%)	0.287 (0.005)	-	0.189 (0.066)	-0.457 (<0.0001)	0.239 (0.013)	-	0.067 (0.493)	-0.273 (0.005)
Lipids (%)	-0.375 (0.0002)	0.189 (0.066)	-	-0.530 (<0.0001)	-0.517 (<0.0001)	0.067 (0.493)	-	-0.501 (<0.0001)
Water (%)	0.103 (0.318)	-0.457 (<0.0001)	-0.530 (<0.0001)	-	0.258 (0.007)	-0.273 (0.005)	-0.501 (<0.0001)	-

BMI; Body Mass Index

THC; Total haemoglobin content

S_t_O^2^; oxygen tissue saturation

In the unadjusted regression analysis with PD as the outcome, BMI showed a significant negative association in both groups (Table 4). S_t_O_2_ and lipids were significantly negatively associated and water significantly positively associated with PD in pre-menopausal women both before and after adjustment for age and BMI. In post-menopausal women these relationships were only observed for lipid and water after adjustment for age and BMI and THC was also positively associated with PD. As age was not associated with PD in both groups in the unadjusted and adjusted analyses it was excluded from further models.

**Table 4:** Unadjusted, adjusted (for age and BMI) and final regression coefficients (β) and standard errors (SEs) for breast tissue optical properties with percent density (square root transformation) for 95 pre- and 107 post-menopausal women.

	**Premenopausal**					**Postmenopausal**				
	**Β**	**SE**	**P-value**	**Ind. R^2^**	**Model R^2^**	**β**	**SE**	**P-value**	**Ind. R^2^**	**Model R^2^**
***Unadjusted***										
Age (years)	0.02	0.05	0.69	< 0.01	-	-0.04	0.03	0.26	0.01	-
BMI (kgm^-2^)	-0.19	0.02	< 0.0001	0.45	-	-0.20	0.03	< 0.0001	0.27	-
THC (*µ*M)	0.08	0.04	0.042	0.04	-	0.17	0.04	< 0.0001	0.15	-
S_t_O_2_ (%)	-0.12	0.02	< 0.0001	0.24	-	-0.02	0.02	0.33	0.01	-
Lipids (%)	-0.10	0.02	< 0.0001	0.30	-	-0.14	0.02	< 0.0001	0.40	-
Water (%)	0.16	0.02	< 0.0001	0.43	-	0.24	0.02	< 0.0001	0.48	-
Amplitude (cm^-1^)	0.08	0.05	0.14	0.02	-	0.21	0.07	0.002	0.09	-
Power	-2.04	0.57	0.0005	0.12	-	-1.59	0.73	0.033	0.04	-
Adjusted*										
THC (*µ*M)	0.05	0.03	0.08	0.02	0.47	0.12	0.0	0.002	0.10	0.36
S_t_O_2_ (%)	-0.05	0.02	0.007	0.04	0.50	-0.02	0.02	0.38	< 0.01	0.31
Lipids (%)	-0.07	0.01	< 0.0001	0.14	0.60	-0.12	0.02	< 0.0001	0.42	0.54
Water (%)	0.10	0.02	< 0.0001	0.14	0.59	0.19	0.02	< 0.0001	0.47	0.55
Amplitude (cm^-1^)	0.01	0.04	0.92	< 0.01	0.46	0.11	0.06	0.08	0.02	0.32
Power	0.22	0.55	0.69	< 0.01	0.46	-0.52	0.69	0.45	< 0.01	0.30
***Final***					0.66					0.65
BMI (kgm^-2^)	-0.12	0.02	< 0.0001	0.45		-0.10	0.03	0.0002	0.03	
S_t_O_2_ (%)	-0.03	0.02	0.06	0.02		-	-	-	-	
Lipids (%)	-0.05	0.01	0.0002	0.14		-0.08	0.02	< 0.0001	0.13	
Water (%)	0.06	0.02	0.008	0.05		0.14	0.02	< 0.0001	0.49	

* Adjusted for age (years) and BMI (kgm^-2^)

**Figure 2:**
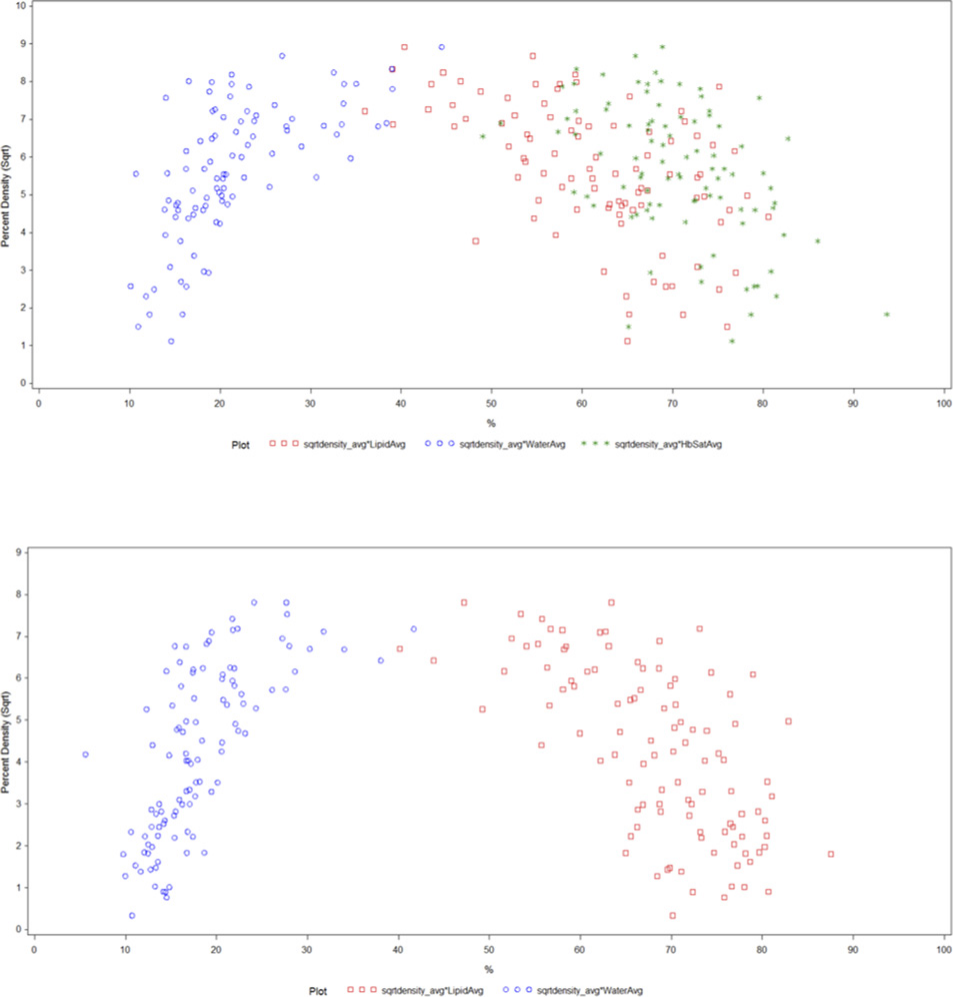
Scatterplot showing the final association of OS chromophore measures [lipid%(red square), water%(blue circle), S_t_O_2_%(green star)] with PD (square root transformed) in pre (top) and post-menopausal women (bottom) adjusted for BMI.

In both groups, in the multivariate analysis (Table 4, Fig. 2a and b), BMI and lipid content both remained significantly inversely related to PD and water content was significantly positively associated with PD, with all three variables explaining ~ 65% of the total variation in PD. However, the association between OS lipid and water content and PD was stronger in post-menopausal women. Among post-menopausal women, water content explained the majority of variation in PD (49% versus 5% in pre-menopausal women), while lipid content explained a similar proportion of the variation in PD in both groups (~13–14%). Among pre-menopausal women, S_t_O_2_ was now only marginally inversely related to PD and BMI explained the majority of the variation in PD, whereas in post-menopausal women it only explained a small proportion (45% versus 3% in post-menopausal women). Once all optical parameters were included in the final model, THC was no longer related to PD in post-menopausal women.

Table 5 presents similar information as Table 4 but for DA. As for PD, S_t_O_2_, and lipids were negatively associated and water positively associated with DA in pre-menopausal women in both unadjusted and age and BMI adjusted analyses. Also similar to PD, in post-menopausal women, THC and water were positively associated and lipids negatively associated with DA both before and after adjustment. Among post-menopausal women, scattering amplitude was also related to DA. Because age and BMI were not associated with DA in both groups in the adjusted analyses they were excluded from further models.

**Table 5:** Unadjusted, adjusted (for age and BMI) and final regression coefficients (β) and SEs for breast tissue optical properties with dense area (square root transformation) for 95 pre-and 107 post-menopausal women.

	**Premenopausal**					**Postmenopausal**				
	**β**	**SE**	**P-value**	**Ind. R^2^**	**Model R^2^**	**β**	**SE**	**P-value**	**Ind. R^2^**	**Model R^2^**
***Unadjusted***										
Age (years)	0.07	0.06	0.24	0.02	-	-0.03	0.04	0.38	< 0.01	-
BMI (kgm^-2^)	-0.003	0.001	0.044	0.04	-	-0.001	0.002	0.42	< 0.01	-
THC (*µ*M)	0.03	0.05	0.46	< 0.01	-	0.18	0.04	< 0.0001	0.14	-
S_t_O_2_ (%)	-0.11	0.03	< 0.0001	0.16	-	-0.04	0.03	0.10	0.03	-
Lipids (%)	-0.06	0.02	0.004	0.09	-	-0.13	0.02	< 0.0001	0.25	-
Water (%)	0.09	0.03	0.004	0.09	-	0.19	0.03	< 0.0001	0.25	-
Amplitude (cm^-1^)	-0.08	0.06	0.19	0.02	-	0.17	0.08	0.03	0.04	-
Power	0.63	0.69	0.36	0.01	-	-0.72	0.82	0.38	< 0.01	-
Adjusted*										
THC (*µ*M)	0.03	0.04	0.54	< 0.01	0.06	0.19	0.05	< 0.0001	0.14	0.15
S_t_O_2_ (%)	-0.10	0.03	0.0001	0.16	0.20	-0.05	0.03	0.09	0.03	0.04
Lipids (%)	-0.06	0.02	0.005	0.09	0.14	-0.14	0.02	< 0.0001	0.30	0.32
Water (%)	0.08	0.05	0.007	0.09	0.13	0.19	0.03	< 0.0001	0.25	0.26
Amplitude (cm^-1^)	-0.07	0.06	0.28	0.02	0.07	0.16	0.08	0.038	0.04	0.05
Power	-0.70	0.68	0.30	0.01	0.07	-0.75	0.83	0.37	< 0.01	0.02
***Final***					0.21					0.37
S_t_O_2_ (%)	-0.10	0.03	0.002	0.16		-	-	-	-	
Lipids (%)	-0.05	0.02	0.019	0.05		-0.10	0.02	< 0.0001	0.30	
Water (%)	-	-	-	-		0.11	0.03	0.001	0.07	

* Adjusted for age (years) and BMI (kgm^-2^)

**Figure 3:**
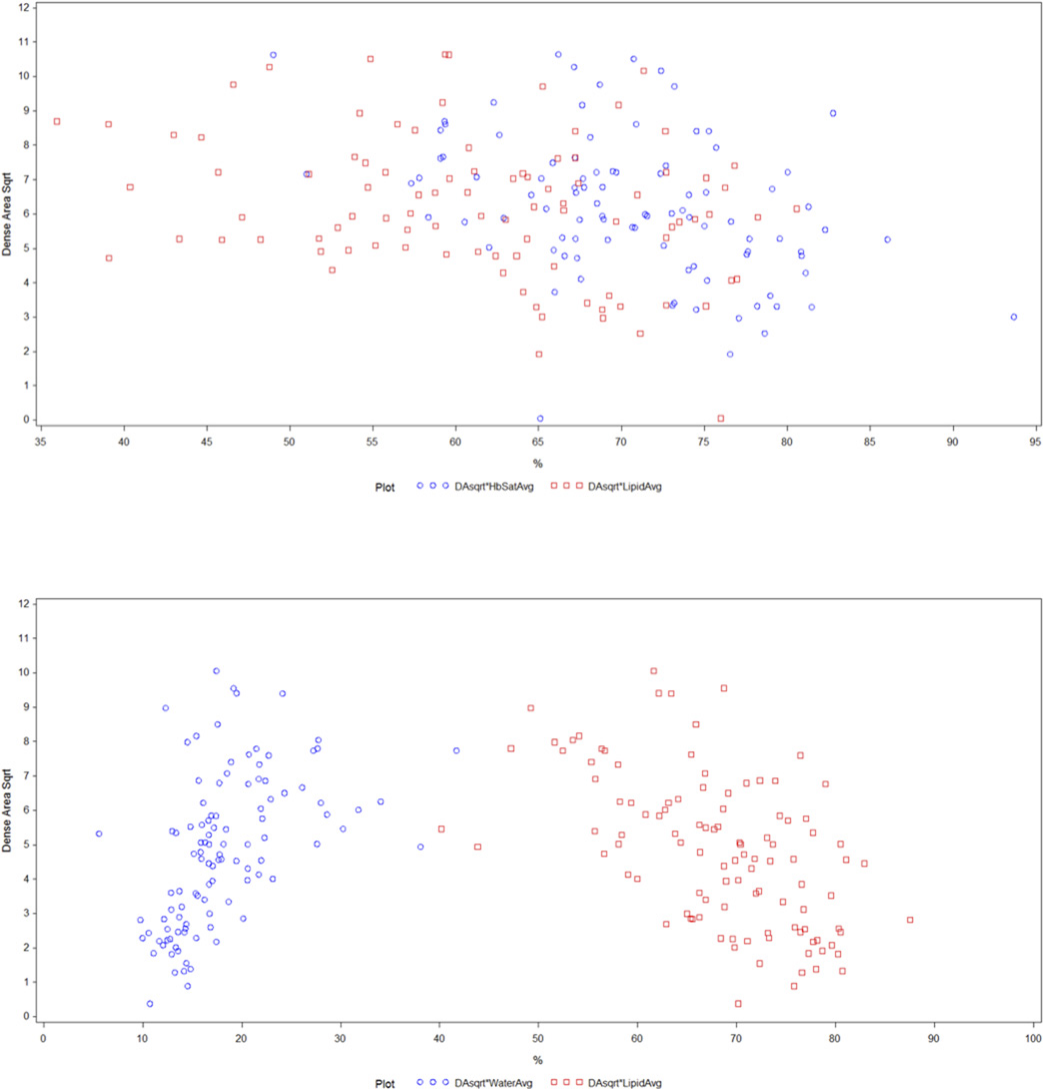
Scatterplot showing the final association of OS chromophore measures [lipid%(red square), water%(blue circle), S_t_O_2_%(green star)] with DA (square root transformed) in pre (top) and post-menopausal women (bottom).

In the multivariate analysis (Table 5, Fig. 3 a and b), lipid content remained significantly and inversely related to DA in both groups, although its association with DA was stronger among post-menopausal women. S_t_O_2_ remained significantly negatively associated with DA among pre-menopausal women. Water content was no longer associated with DA in the pre-menopausal group, but remained positively associated with DA in post-menopausal women. Overall, OS breast content accounted for a larger proportion of the total variation in DA in post-menopausal women (~ 37%) compared to pre-menopausal women (~ 21%). Once all optical parameters were included in the final model, THC and scattering amplitude, were no longer related to DA in post-menopausal women.

## DISCUSSION

This study expands on our previous work on OS and mammographic density [19] by examining the association between OS-derived content of water, lipid, THC, S_t_O_2_, scattering amplitude and power in the female breast and quantitative mammographic features among 95 pre- and 107 post-menopausal women. Consistent with expectation, we found greater OS-derived lipid content in low density tissue and higher OS-derived water content in high density tissue. We also observed lower oxygen saturation to be associated with dense tissue in younger women. OS is a potential alternative non-invasive method of assessing breast tissue risk capturing similar as well as potentially complementary information to mammographic density.

The associations we observed between age and BMI and OS parameters among pre- and post-menopausal women were as expected based on previous work [21–29]. Increasing BMI, indicative of higher overall lipid content, was associated with increased lipid content in breast tissue, but decreased water content, reflecting the lower amount of fibroglandular tissue in women with higher BMI [38]. Interestingly, age was not associated with OS parameters in pre-menopausal women, only post-menopausal women, in particular THC and lipid content. This is likely related to the atrophy of glandular tissue consequent with changing hormone levels associated with menopause, resulting in less proliferative tissue (hence decreased THC) and the replacement by fatty tissue, resulting in increased lipid content [38].

The values we obtained for OS-derived breast content are comparable to previously published optical studies examining breast tissue in pre- and post-menopausal women [23, 27, 29]. O’Sullivan et al. [29] noted a mean lipid content of 67.0% and a mean water content of 24.4% in pre-menopausal women (40.8 ± 5.0 years). In post-menopausal women (57.6 ± 7.2 years) they observed a mean lipid content of 74.0% and mean water content of 16.6% [29], while Cerussi and colleagues (56 ± 5 years) noted slightly lower lipid and water contents of 67.5% and 10.3%, respectively using a reflectance probe [23]. Our calculated values for THC and S_t_O_2_ in pre-menopausal women are slightly lower than those described by O’Sullivan at al. (15.7 versus 24.2*µ*M and 70.5 versus 76.8%, respectively) [29]. Among post-menopausal women, our THC values are also lower than other reported mean values (12.4 versus 14.4 *µ*M to 21.1*µ*M), while our S_t_O_2_ values fall within the lower end of the reported range (68.5 versus 68.3% to 83.7%) [23, 27, 29].

Few studies have examined the association between breast optical content and mammographic density [26–29, 39] and none have considered how these associations may differ according to menopausal status. Suzuki et al [39] performed time resolved spectroscopy measurements on 30 Japanese women (48.9 ± 14.3 years) with normal breasts and found that overall light absorption was significantly higher in women with mammographic patterns of DY or P2 compared to N1 or P1 (Wolfe’s classification); overall scattering, however, was not different between these two groups. Srinivasan et al. [26, 27], using NIR tomography in healthy breasts of 60 women (57 ± 10.8 years), found that both S_t_O_2_ and scatter power could discriminate heterogeneously dense and extremely dense breasts (BIRADS 3 and 4, respectively) from almost entirely fatty breast (BIRADS 1). They also observed that water content (%) for BIRADS 1 and 2 type breasts was similar, but statistically lower than BIRADS 3 and 4. Taroni et al. [28] employed time-resolved transmittance spectroscopy on 49 women (51.4 ± 11.5 years) with normal breasts and found water content to be significantly higher and lipid content to be significantly lower in breasts classified as BIRADS 4 versus 3, and BIRADS 3 versus 2; trends for increasing THC, scatter power and amplitude were also noted with increasing density categories. A recent study by O’Sullivan and colleagues measured the contralateral normal breast of 28 women (47.4 ± 10.2 years) with breast cancer using diffuse optical spectroscopic imaging (DOSI) and MRI before neoadjuvant chemotherapy [29]. They found a significant difference in THC between BIRADS categories 3 and 4 and a significant difference in water between BIRADS categories 2 and 4; lipid concentration decreased with increasing BIRADS categories and approached significance. Water content and THC from DOSI also correlated positively with breast density calculated from MRI (r = 0.84 and r = 0.60, respectively) while lipid content correlated negatively with MRI derived breast density (r = -0.71).

The final associations that we observed between OS breast content of lipid, water and S_t_O_2_ and PD and DA among pre- and post-menopausal women are in agreement with previous optical studies and also reveal variations in breast tissue physiology by menopausal status. Due to changes in hormonal exposure consequent with age and menopause, atrophy of glandular tissue occurs with concomitant replacement by adipose tissue [38]. Consequently the proportion of fibroglandular tissue relative to adipose tissue is, on average, smaller in post-menopausal women [38]. Among both groups of women OS lipid content was inversely related to PD, even after adjustment for BMI, suggesting that OS captures additional information about the fat component of PD, beyond that captured by BMI alone. However, among post-menopausal women, lipid content was more strongly inversely related to DA than it was among pre-menopausal women, suggesting that the association between lipid and PD in pre-menopausal women is likely through its relationship with the proportion of the non-dense (i.e. fatty) component of the breast.

Glandular tissue has higher water content than adipose tissue [23, 26, 40–41]; it is also more vascularized and metabolically active [23, 26]. As a result, higher water content and lower S_t_O_2_ is expected in high density breast tissue [23, 26]. In agreement with these observations we found that water content was positively related to PD in both groups, while it was only related to DA in post-menopausal women. Conversely, among pre-menopausal women, S_t_O_2_ was marginally inversely related to PD, but strongly related to DA. These differences are consistent with more glandular and proliferative tissue in the breasts of younger women prior to menopause, whereas after menopause, atrophy of glandular tissue (DA) and its replacement by fatty tissue (non-DA) primarily characterizes breast tissue composition in older women [38].

There are several strengths of this study including a larger sample of women than earlier studies relating optical content to mammographic density measures [26–29, 39]. Our increased sample size also allowed us to examine the relationship between OS-derived measures of the breast and quantitative measures of both PD and DA by menopausal status, which has not been done previously. While the OS technology utilized here is far less complex than the time-resolved measurements used by other studies [23–24, 26–29] and hence easier to implement in a larger study population, there are some limitations. The reproducibility of the OS chromophore measures has not yet been assessed in women measured at multiple time points. Measurements from some women had to be excluded as the true diffuse transmission spectra could not be approximated by the fitting routine with sufficient confidence resulting in a larger overall error in the estimation of their breast tissue content. Also, there was a considerable fraction of breast tissue composition that we could not account for, likely due to additional components that were not included (e.g. collagen) and because of photon losses at the breast boundary, even at the distal position. Collagen content may be of interest with respect to its relationship to mammographic density [42]; however, it is very difficult to estimate because its spectral shape is relatively flat and overlaps considerably with other components, particularly water and the tail end of the scattering function. To separate these two components, time resolved measures would be required. It is also challenging to validate the estimated component measures; but it is reassuring that our results are consistent with expectations given our knowledge of breast physiology and the results of earlier optical studies. Although our sample size was large relative to other studies, an even larger sample size could be even more informative. Also, we did not have information on a number of known hormonal risk factors for breast cancer and we will be exploring the relationship between the OS chromophore measures and hormonal risk factors in other studies.

## CONCLUSIONS

In this study, OS-derived measures of water, lipid and S_t_O_2_ were related to PD and DA in pre- and post-menopausal women. Given our observed relationships, OS measures may prove useful as intermediate markers in studies of breast cancer etiology and prevention. They may also be useful for assessing physiologic tissue differences related to breast cancer risk and/or measuring differences from cumulative exposure to risk factors known to modulate breast cancer risk, particularly in younger women where mammography is not an option or to stratify access to mammography when infrastructure is limited. OS is a fast method of breast assessment with no radiation exposure that may prove useful in a clinical setting, although further developmental work is needed. We are continuing to develop and assess this approach with the goal of developing a clinically useful tool. The strong relationship with an established risk assessment marker such as mammographic density is encouraging.
